# *Pichia pastoris* versus *Saccharomyces cerevisiae:* a case study on the recombinant production of human granulocyte-macrophage colony-stimulating factor

**DOI:** 10.1186/s13104-017-2471-6

**Published:** 2017-04-04

**Authors:** Anh-Minh Tran, Thanh-Thao Nguyen, Cong-Thuan Nguyen, Xuan-Mai Huynh-Thi, Cao-Tri Nguyen, Minh-Thuong Trinh, Linh-Thuoc Tran, Stephanie P. Cartwright, Roslyn M. Bill, Hieu Tran-Van

**Affiliations:** 1 Faculty of Biology and Biotechnology, University of Science, VNU-HCM, Ho Chi Minh, Vietnam; 2 School of Life and Health Sciences, Aston University, Birmingham, UK

**Keywords:** *S. cerevisiae*, *P. pastoris*, rhGM-CSF, Fermentation, Bioactivity, TF-1 cell

## Abstract

**Background:**

Recombinant human granulocyte-macrophage colony-stimulating factor (rhGM-CSF) is a glycoprotein that has been approved by the FDA for the treatment of neutropenia and leukemia in combination with chemotherapies. Recombinant hGM-CSF is produced industrially using the baker’s yeast, *Saccharomyces cerevisiae*, by large-scale fermentation. The methylotrophic yeast, *Pichia pastoris*, has emerged as an alternative host cell system due to its shorter and less immunogenic glycosylation pattern together with higher cell density growth and higher secreted protein yield than *S. cerevisiae*. In this study, we compared the pipeline from gene to recombinant protein in these two yeasts.

**Results:**

Codon optimization *in silico* for both yeast species showed no difference in frequent codon usage. However, rhGM-CSF expressed from *S. cerevisiae* BY4742 showed a significant discrepancy in molecular weight from those of *P. pastoris* X33. Analysis showed purified rhGM-CSF species with molecular weights ranging from 30 to more than 60 kDa. Fed-batch fermentation over 72 h showed that rhGM-CSF was more highly secreted from *P. pastoris* than *S. cerevisiae* (285 and 64 mg total secreted protein/L, respectively). Ion exchange chromatography gave higher purity and recovery than hydrophobic interaction chromatography. Purified rhGM-CSF from *P. pastoris* was 327 times more potent than rhGM-CSF from *S. cerevisiae* in terms of proliferative stimulating capacity on the hGM-CSF-dependent cell line, TF-1.

**Conclusion:**

Our data support a view that the methylotrophic yeast *P. pastoris* is an effective recombinant host for heterologous rhGM-CSF production.

**Electronic supplementary material:**

The online version of this article (doi:10.1186/s13104-017-2471-6) contains supplementary material, which is available to authorized users.

## Background

Human granulocyte-macrophage colony-stimulating factor (hGM-CSF), a glycosylated cytokine, plays a vital role in proliferation and differentiation of granulocytes and macrophages from bone marrow progenitor cells [1]. hGM-CSF also enhances cytotoxicity of macrophages and monocytes towards tumor cells [2], as well as tumor presentation of dendritic cells [1, 3]. Due to its stimulatory capacity on hematopoietic stem cells, recombinant hGM-CSF (rhGM-CSF) is recommended for therapeutic use in combination with chemo- or radio-therapy for cancer or transplantation patients [4].

hGM-CSF comprises 127 amino acids. The presence of 2 *N*- and 4 *O*-glycosylation sites leads to different glycoforms with molecular weights ranging from 14 to 60 kDa [3]. The glycosylation of the protein affects its pharmacokinetics, in vivo half-life, immunogenicity and cytotoxicity [3].

rhGM-CSF is produced from bacteria, baker yeast or mammalian hosts [5]. rhGM-CSF produced in *Escherichia coli* is biologically active but unstable in human plasma. It induces an immune reaction because N-formyl methionine (fMet) is its first amino acid. rhGM-CSF produced in mammalian hosts has a similar glycosylation pattern to the native human protein, but production rates are slow [6, 7]. The product from baker’s yeast, *Saccharomyces cerevisiae*, is glycosylated and approved by the FDA for the treatment of neutropenia and leukemia in combination with chemo- or radio-therapy for cancer or transplantation patients. The drawbacks of using *S. cerevisiae* are hyper-glycosylated products and low cell density growth.

The methylotrophic yeast *Pichia pastoris* has emerged as an alternative host due to its shorter and less immunogenic glycans, higher density cell growth and higher secreted protein yields than *S. cerevisiae*. In this study, we show that *P. pastoris* secretes a higher yield of more active recombinant rhGM-CSF than *S. cerevisiae*.

## Methods

### *In silico* codon optimization

hGM-CSF coding sequences were from Gene Bank (Accession M11220). *In silico* codon optimization was done using DNA 2.0 software with *P. pastoris* and *S. cerevisiae* codon tables and optimized parameters [8]. The optimization evaluation was done via http://www.gcua.schoedl.de with low (<20%) frequency codon usage displayed as hatched bars and very low (<10%) frequency codon usage as white bars.

### Expression of rhGM-CSF in *P. pastoris* and *S. cerevisiae*

Recombinant *P. pastoris* and *S. cerevisiae* rhGM-CSF clones were expressed as described previously [9, 10]. The expression of the rhGM-CSF was analyzed by SDS-PAGE and immuno blot probed with an antibody against hGM-CSF (LifeSpan BioScience). Briefly, recombinant *S. cerevisiae* strain BY4742/*hgm*-*csf* was cultured in selective CSM-ura medium (6.7 g/L yeast nitrogen base with ammonium sulphate, 0.1 M sodium phosphate, supplemented with amino acids lacking uracil [11]) supplemented with 2% glucose until the OD_600_ of the culture was 0.5–1. The cells were then harvested and transferred into CSMG-ura medium (CSM supplemented with 20 g/L galactose) with shaking at 230 rpm at 30 °C. The pH 6.0 of each medium was stabilized by 0.1 M sodium phosphate buffer. The sample supernatants were collected after 72 h of induction and stored for further analysis.

Recombinant *P. pastoris* X33::*hgm*-*csf* was grown in 10 mL BMGY medium (2% peptone, 1% yeast extract, 0.34% yeast nitrogen base, 1% ammonium sulfate, 1% glycerol, and 0.4 mg/L biotin, buffered with 1/10 volume of pH 6.0 potassium phosphate buffer) at 30°C with constant shaking at 250 rpm until the culture reached an OD_600_ of 2–4. The cells were harvested and re-suspended in 10 mL BMMY (0.5% methanol is substituted for 1% glycerol in BMGY) with the same growth conditions. For induction, methanol was added every 24 h to a final concentration of 0.5%. After 72 h, culture supernatants were collected by centrifugation for further analysis.

### Fermentation of recombinant *P. pastoris* and *S. cerevisiae*

Recombinant yeast *S. cerevisiae* strain BY4742/*hgm*-*csf* was batch-cultured in selective CSM(-ura) medium, pH 5.0 supplemented with 2% glucose until the culture reached an OD_600_ of 0.5 to 1, and then transferred into 3.18 L YP medium to a final OD_600_ of 0.5 in a 5 L LiFlus-GX fermenting vessel system (Biotron). Others growth parameters were temperature at 30 °C, aeration rate 1.5 vvm (through a filter), and agitation speed 720 rpm. Every 24 h, 40 g/L galactose was added at a rate of 6.7 mL/h. Samples were harvested at every 4 h and analyzed. Growth of the yeast cultures was monitored by optical density measurements at 600 nm. Protein secretion in the fermented medium was analyzed by SDS-PAGE and the protein concentration was determined using Bradford’s method [12]. The supernatant was stored at −20 °C for later purification.

For *P. pastoris* expression [13], a flask containing 150 mL BMGY containing 0.4 µg/mL biotin was inoculated from a frozen glycerol stock. The inoculum seed flask was grown at 30 °C, 250 rpm, and 24 h until OD_600_ = 2–6. A sterilized fermenter containing 2.5 L Fermentation Basal Salts medium supplemented with 4% glycerol and 11 mL PTM_1_ trace salts was prepared, the pH adjusted to 5.0 with ammonium hydroxide, the temperature set to 30 °C, agitation at 750 rpm and aeration to 5.0 vvm air. The culture in the inoculum seed flask was completely transferred into the fermenter in which the batch culture was grown for 24 h, until the glycerol was completely consumed. Glycerol feeding was initiated for about 4 h at a feed rate to 18.15 mL/h 50% w/v glycerol containing 1.2% v/v PTM_1_ trace salts. Methanol feeding was then initiated for 72 h at a feed rate of 100% methanol containing 1.2% v/v PTM_1_ trace salts as follows: 9 mL/h for 3 h; 18 mL/h for 3 h and then 27 mL/h. The supernatant was analyzed by SDS-PAGE every 4 h and the protein concentration was determined using Bradford’s method [12]. The supernatant was stored at −20 °C for later purification.

### Ion exchange chromatography and hydrophobic interaction chromatography purification of recombinant rhGM-CSF

rhGM-CSF was purified using hydrophobic interaction chromatography. A HiPrep Phenyl Fast Flow column was equilibrated in buffer A [(NH_4_)_2_SO_4_ 2 M, CH_3_COONa 20 mM, pH 5.0]. Approximately 20 mL of filtered supernatant was loaded onto the column at 1 mL/min. The column was then washed with 10 column volumes of buffer B. The bound protein was subsequently eluted with buffer B (Na_2_HPO_4_ 10.14 mM; KH_2_PO_4_ 1.76 mM; NaCl 136.89 mM; KCl 2.68 mM, pH 7.4) in a step-wise manner, ranging from 5, 10, 20, 40, 60 and 100% buffer B. The target fractions were identified by SDS-PAGE. The protein was eluted maximally at 10 to 20% buffer B.

rhGM-CSF was alternatively purified using anion exchange chromatography at pH 7.5. A HiTrap Q Sepharose Fast Flow column was equilibrated in 50 mM Tris-HCl. Approximately 20 mL of filtered and desalted supernatant was loaded onto the column at 1 mL/min. The column was then washed with 10 column volumes of 50 mM Tris-HCl. The bound protein was subsequently eluted in a step-wise manner with the NaCl concentration optimized over a range of 0–1 M NaCl in 50 mM Tris-HCl. The target fractions were identified by SDS-PAGE. The protein was eluted maximally at 0.1–0.2 M NaCl.

### Bioactivity of purified rhGM-CSF

Purified rhGM-CSF from each yeast species was added to 1 × 10^5^ cells/mL of TF-1 (a GM-CSF dependent cell line) in RPMI plus 10% FBS to obtain a final rhGM-CSF concentration ranging from 2 × 10^4^ to 2 × 10^−1^ pg/mL. 100 µL of each combination was aliquoted into a 96-well plate and cultured for 72 h in a 5% CO_2_ incubator. The proliferation of TF-1 cells was measured by using a CCK-8 kit at OD 450 nm according to the manufacturer’s instructions. Crude supernatants from *P. pastoris* and *S. cerevisiae* served as controls. LU (Laboratory unit) was calculated as follows:

$${\text{LU}}\,({\text{units}}/{\text{mg}}) = \frac{{1 \times 10^{9} }}{{{\text{ED}}50}}$$

## Results

### *In silico* optimization removes low frequency codon usage in both yeasts

**Fig. 1:**
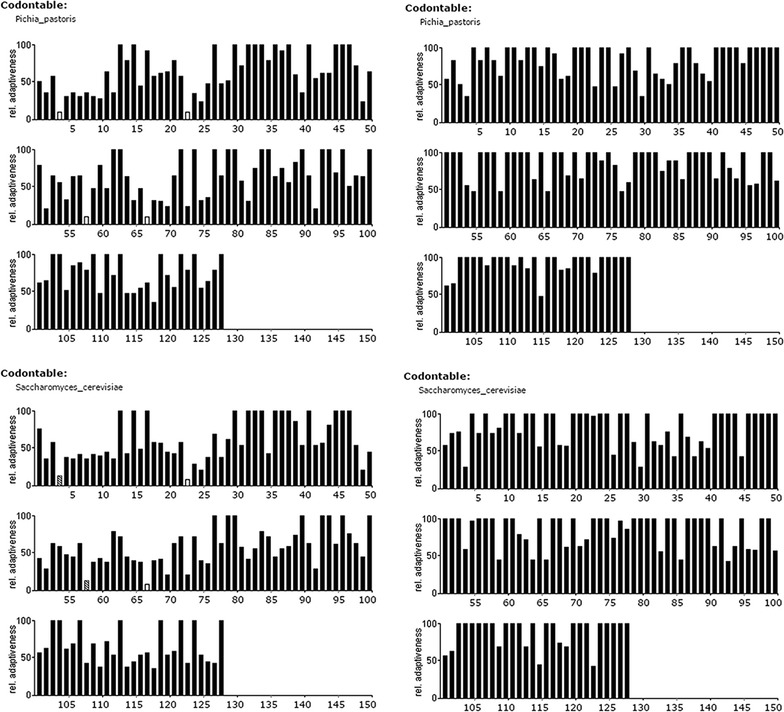
Relative adaptiveness (frequency of codon usage) of the sequence encodinghGM-CSF prior (*left column*) and post (*right column*) *in silico* optimization for expression in *P. pastoris* and *S. cerevisiae*. Low (<20%) frequency codon usage is shown *in hatched bars* while very low (<10%) frequency codon usage is shown in *white bars*

In contrast to the native human sequence, both *in silico*-optimized sequences show a high relative adaptiveness (Fig. 1, right column). These low (<20%, hatched bars) or very low (<10%, white bars) frequency codons were substituted for higher frequency codons. The optimization step did not introduce restriction enzyme(s) to the optimized sequences (data not show) which facilitated the subsequent cloning steps.

### Differences in the expression profile of rhGM-CSF in the two yeast species

**Fig. 2:**
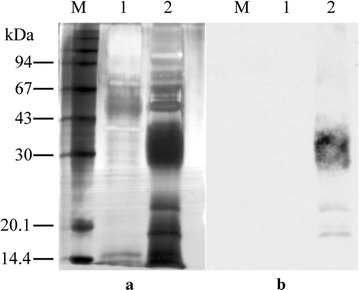
The expression of rhGM-CSF in *P. pastoris* analyzed by SDS-PAGE (**a**) and immunoblot probed with an anti-hGM-CSF antibody (**b**). M, molecular mass markers; 1, *P. pastoris* X33 culture supernatant 72 h post-induction with methanol; 2, *P. pastoris* X33::*hgm*-*csf*culture supernatant 72 h post-induction; 50 µL crude supernatant was precipitated and loaded per lane

**Fig. 3:**
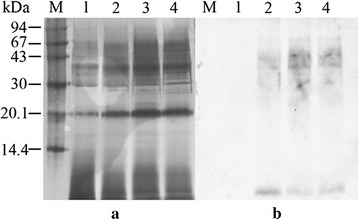
The expression of rhGM-CSF in *S. cerevisiae* analyzed by SDS-PAGE (**a**) and immunoblot probed with an anti-hGM-CSF antibody (**b**).* M* molecular markers;* 1* supernatant from *S. cerevisiae* BY4742 72 h post-induction with galactose;* 2*,* 3* and* 4*, supernatant from *S. cerevisiae* BY4742/pYES2-*hgm*-*csf* induced with galactose after 24, 48 and 72 h, respectively 50 µL crude supernatant was precipitated and loaded per lane

The expression profile of rhGM-CSF was analyzed using SDS-PAGE and probed with a specific antibody against hGM-CSF. The induction of *P. pastoris* with methanol led to the expression of several proteins (Fig. 2). Probing with a specific antibody showed the majority of the expressed rhGM-CSF ranged in size from 28–43 kDa with two minor bands at 17.8 and 24 kDa. In contrast, following induction of recombinant *S. cerevisiae* cells with galactose, there was no difference compared to control cells (Fig. 3). Probing with a specific antibody showed the majority of expressed rhGM-CSF ranged in size from 30 to 67 kDa.

### Recombinant hGM-CSF is highly secreted from *P. pastoris*

**Fig. 4:**
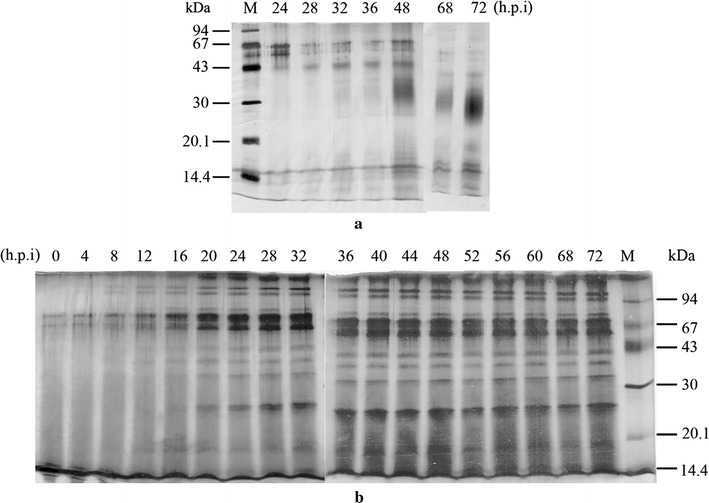
The secreted expression of rhGM-CSF analyzed by SDS-PAGE; hours post induction (h.p.i) are indicated for *P. pastoris* X33::*hgm*-*csf* induced with methanol (**a**) or *S. cerevisiae* BY4742/pYES2-*hgm*-*csf* induced with galactose (**b**). *M* molecular mass markers; 50 µL crude supernatant was precipitated and loaded per lane

**Fig. 5:**
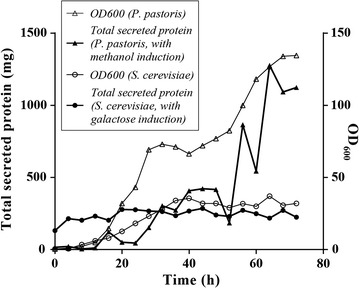
OD and total secreted proteins in supernatants of *P. pastoris* X33::*hgm*-*csf* induced with methanol or *S. cerevisiae* BY4742/pYES2-*hgm*-*csf* induced with galactose was monitored over 72 h time course with 4 h intervals. Data are representative of three independent experiments

Time-course expression showed an accumulation of rhGM-CSF (Fig. 4). In the last 72 h, the secreted protein/OD_600_ ratio was dramatically increased in *P. pastoris*. The cultures grew to a very high density of 134 OD units over the time course compared to 32 OD units for *S. cerevisiae* (Fig. 5). Consequently, this led to a higher total secreted protein yield per mL culture medium at 72 h post-induction (285 mg for *P. pastoris* vs 64 mg for *S. cerevisiae*).

### Ion exchange chromatography is more efficient in recovery of rhGM-CSF than hydrophobic interaction chromatography

**Fig. 6:**
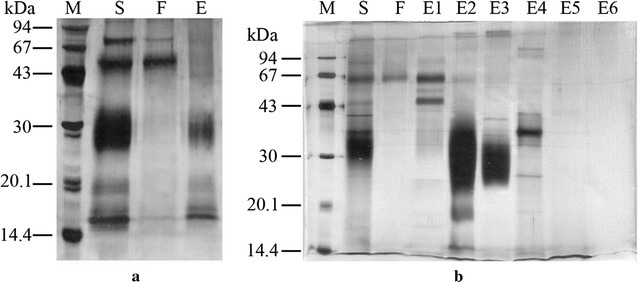
Purification of rhGM-CSF from culture supernatants of *P. pastoris* X33::*hgm*-*csf* induced with methanol analyzed by SDS-PAGE. **a** Hydrophobic interaction chromatography. *M* molecular mass markers, *S* supernatant after 72 h, *F* column flow-through, *E* eluted fraction. **b** Ion exchange chromatography. *M* molecular mass markers, *S* supernatant after 72 h, *F* column flow-through, *E1*–*6* fractions eluted with NaCl (50, 100, 200, 400, 600 and 1000 mM, respectively)

Recombinant hGM-CSF produced in *P. pastoris* was eluted maximally at 10–20% buffer phosphate. The purity was 85.2% and the recovery yield was 53.8% (Fig. 6 and Table 1). In contrast, using ion exchange chromatography, rhGM-CSF was eluted with 10 and 20% elution buffer; the purity ranged from 92 to 95%, and the recovery yield was 36–56% (Fig. 6 and Table 1). Collectively, ion exchange chromatography was more efficient in recovery of rhGM-CSF than hydrophobic interaction chromatography. A similar result was also obtained for rhGM-CSF produced in *S. cerevisiae* (data not shown and Table 1).

**Table 1:** Purification yield of rhGM-CSF from *P. pastoris* and *S. cerevisiae* using IEC with % elution buffer or HIC

Sample	GM-CSF from *P. pastoris* (IEC) 10%	GM-CSF from *P. pastoris* (IEC) 20%	GM-CSF from *P. pastoris* (HIC)	GM-CSF from *S. cerevisiae* (IEC) 10%	GM-CSF from *S. cerevisiae* (IEC) 20%	GM-CSF from *S. cerevisiae* (HIC)
Purity (%)	92.7	95.2	85.2	95.6	64.22	42.9
Purification yield (%)	56.9	35.9	53.8	21.0	30.8	0.5

### Recombinant hGM-CSF produced from *P. pastoris* shows high bioactivity on a GM-CSF-dependent cell line

**Fig. 7:**
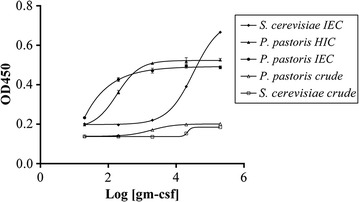
Dose response curve for purified rhGM-CSF from *P. pastoris* and *S. cerevisiae* using ion exchange chromatography (IEC) or hydrophobic interaction chromatography (HIC) on the GM-CSF dependent cell line TF-1

The purity of a sample strongly affects its bioactivity. Thus to precisely evaluate bioactivity, rhGM-CSF produced from both yeasts was only tested with a purity above 80%. These samples were evaluated using a GM-CSF-dependent cell line, namely TF-1. The response curves showed a typical sigmoidal cytokine response. To compare the responses, effective dose 50 (ED_50_) (pg/mL) and laboratory unit (LU) (units/mg) were calculated from each curve as shown in Table 2. The ED_50_ of purified rhGM-CSF increased on going from protein produced using *P. pastoris* and IEC, *P. pastoris* and HIC, *S. cerevisiae* and IEC to *S. cerevisiae* and HIC. Notably, purified rhGM-CSF from *P. pastoris* using IEC had higher purity than protein from *P. pastoris* using HIC (Fig. 7).

**Table 2:** Bioactivity of purified rhGM-CSF from *P. pastoris* and *S. cerevisiae* using IEC or HIC

	ED50 (pg/mL)	LU (units/mg)	Response curve range (OD unit)
GM-CSF from *P. pastoris* (IEC)	84.5	11.8 × 10^6^	0.26
GM-CSF from *P. pastoris* (HIC)	198.7	5.0 × 10^6^	0.32
GM-CSF from *P. pastoris* (crude)	1876	0.53 × 10^6^	0.06
GM-CSF from *S. cerevisiae* (IEC)	27,880	0.36 × 10^5^	0.49
GM-CSF from *S. cerevisiae* (crude)	37,238	0.27 × 10^5^	0.05

## Discussion

Four publications describe rhGM-CSF expression in three different yeast systems, including the well-known yeasts *S. cerevisiae*, *P. pastoris* and *Yarrowia lipolytica* [14–17], but none conducts a head-to-head comparison. In the present study, we used an *in silico*-optimized synthetic hGM-CSF gene in two yeast expression vectors. The expression patterns were similar to those previously published [14, 15, 17].

Using fed-batch fermentation, we obtained yields of rhGM-CSF of 9.5 and 180.3 mg/L from *S. cerevisiae* and *P. pastoris* respectively. The latter is higher than has been previously described [17], possibly because we used single-copy rather than high-copy integration. All published protocols on the purification of rhGM-CSF combine two different purification steps to get above 95% purity [14–17]. In our study, we used a one-step procedure to obtain greater than 95% purity.

The activity of purified rhGM-CSF from *S. cerevisiae* on TF-1 cells was 0.36 × 10^5^ LU/mg, which is lower than that of the commercially available drug Leukine (rhGM-CSF). The discrepancy could be due to the glycosylation moiety added to rhGM-CSF [3] which in turn could have an effect on its bioactivity. The drug Leukine contains a non-glycosylated rhGM-CSF in contrast to our purified, glycosylated rhGM-CSF. Purified rhGM-CSF from *P. pastoris* had an activity of 1.2x10^7^ LU/mg. This activity is almost equal to the standard sample announced by WHO [18] and is two times higher than that of Leukine. Compared to previous data [17], our purified rhGM-CSF is 15 times less active. When compared with our *E. coli*-derived rhGM-CSF (non-glycosylated), our data show that rhGM-CSF is 1.5 times lower than that of *E. coli* origin (unpublished data). With the same purity, rhGM–CSF from *S. cerevisiae* was 327 times less active than that from *P. pastoris*.

## Conclusion

In this study, we have been able to compare the pipeline from gene to recombinant protein between *S. cerevisiae* and *P. pastoris*. Our results suggest that rhGM-CSF expressed in *P. pastoris* is more active than the same protein from *S. cerevisiae*. To our knowledge, the data presented here are the first head-to-head comparison of *S. cerevisiae* and *P. pastoris* as host cells for rhGM–CSF production.

## Additional files

**Additional file 1.** Results of fed-batch fermentation process of *P. pastoris* X33::*hgmcsf* and *S. cerevisiae* BY4742/pYES2-*hgmcsf.*

**Additional file 2.** Results of bioactivity analysis of purified hGM-CSF derived from *P. pastoris* X33::*hgmcsf* and *S. cerevisiae* BY4742/pYES2-*hgmcsf.*

## Abbreviations

rhGM-CSF: recombinant human granulocyte-macrophage colony-stimulating factor
FDA: Food and Drug Administration
TF-1: human erythroleukemic cell line
SDS-PAGE: sodium dodecyl sulfate-polyacrylamide gel electrophoresis
OD: optical density
LU: laboratory unit
ED50: effective dose 50
IEC: ion exchange chromatography
HIC: hydrophobic interaction chromatography
WHO: World Health Organization
